# A Review of Three New Anti-interleukin-5 Monoclonal Antibody Therapies for Severe Asthma

**DOI:** 10.7759/cureus.3216

**Published:** 2018-08-27

**Authors:** Vivian C Agumadu, Kamleshun Ramphul, Stephanie G Mejias, Ruhi Sonaye, Shaheen Sombans, Petras Lohana

**Affiliations:** 1 Medicine, International University of the Health Sciences School of Medicine, Basseterre, KNA; 2 Pediatrics, Shanghai Jiao Tong University School of Medicine/Shanghai Xin Hua Hospital, Shanghai, CHN; 3 Pediatrics, The University Iberoamericana Unibe School of Medicine/Robert Reid Cabral Children's Hospital, Santo Domingo, DOM; 4 Bharati Vidyapeeth Deemed University Medical College and Hospital, Thane, IND; 5 Internal Medicine, Bharati Vidyapeeth Deemed University Medical College and Hospital, Pune, IND; 6 Medicine, Liaquat University of Medical and Health Sciences Hospital, Karachi, PAK

**Keywords:** monoclonal antibody, asthma

## Abstract

Asthma is a chronic respiratory condition that is characterized by reversible airflow obstruction. Interleukin-5 (IL-5) is involved in the pathophysiology of the disease and drugs targeting IL-5 have been studied for years as a possible treatment option for severe asthma. In this review, the authors searched PubMed for major drug therapies and clinical trials against IL-5. A total of 29 articles met the criteria for selection and were shortlisted; of these, 10 papers were on benralizumab, 14 on mepolizumab, and five on reslizumab.

The three drugs proved to be safe and efficacious for patients with severe asthma, leading to decreased rates of asthma exacerbations, lowered levels of eosinophils, and improved pulmonary functions in various studies. Patients also reported an improvement in the quality of life. The side effects of these three drugs were mild and no deaths directly linked to the drug were reported. However, longer duration studies are required to draw firm and strong conclusions on the safety of these therapeutic agents.

## Introduction and background

Asthma is a chronic respiratory condition that is characterized by reversible airflow obstruction. There are a number of environmental and genetic factors associated with the development of the condition. It causes a significant economic burden on society, affecting more than 334 million people worldwide. Asthma is estimated to cause 250,000 deaths annually and consumes billions of dollars in treatment. The global impact of asthma and the prevalence of the disease is constantly increasing [1].

Treatment plans for asthma vary according to the severity of the condition and the response to previous therapy. The aim is to achieve control and prevent future exacerbations of the disease. The treatment of choice for mild cases of asthma and for intermittent asthma involves treatment with a short-acting beta agonist such as albuterol. If the asthma is persistent, the physician may choose to alter the treatment options in a stepwise manner by adding low dose inhaled corticosteroids (ICS). The doses of ICS can be further increased depending on the severity and failure to show any signs of improvement by the patients. Other treatment options include the use of leukotriene receptor antagonists (LTRAs) such as montelukast or zafirlukast. The use of theophylline or zileuton may also be considered. Montelukast is also a treatment option for exercise-induced asthma and has been approved for use in children as young as one year of age while zafirlukast can be used in children up to the age of seven. For cases of steroid-resistant asthma, omalizumab is also a possible treatment option [2].

Severe asthma is classified as asthma with symptoms that persist and are uncontrolled despite a high dose of ICS and a second controller and/or systemic corticosteroids [3]. An estimated 5%-10% of asthma patients are classified as severe asthma while recent data suggests that the number might be lower than five percent [4]. Two-thirds of patients with severe asthma suffer from late-onset asthma with a higher female to male ratio [5]. Uncontrolled asthma presents with at least one of the following: frequent severe exacerbations, serious exacerbations, or airflow limitation with persistent low forced expiratory volume in one second (FEV1) after appropriate bronchodilation and/or poor symptom control [6].

Since the pathophysiology of asthma involves multiple immunological cells and cytokines, recent therapeutic advances over the past three decades focused on identifying anti-cytokine and monoclonal antibody therapies. Eosinophils can release their pro-inflammatory mediators, such as major basic proteins, cytokines, and chemokines that potentiate the lung injury. Interleukin-5, which is derived from type two helper cells (TH2), plays a major role in the development and release of eosinophils. It has been considered as one of the therapeutic targets for preventing and treating asthma [7-8]. The aim of this review is to provide a concise discussion of the various monoclonal therapies against IL-5 that are available for the treatment of asthma.

## Review

Methods

The authors searched PubMed to identify major drug therapies and clinical trials against IL-5. The search was restricted to articles published from the first of January 2000 to the first of May 2018. The results were further limited to articles published in English. Studies were independently reviewed by each author prior to preparing a pooled analysis.

Results

The authors found 29 articles of interest; of these, 10 papers were on benralizumab, 14 on mepolizumab, and five on reslizumab. The selection was limited to clinical studies published in English and investigating primarily the changes in clinical presentations of patients with severe asthma and/or eosinophil levels. We further discussed any difference in statistical significance in the results that were found among the selected studies in the review.

Discussion

The authors focused on three main therapies against IL-5 that have undergone several clinical trials. A brief understanding of the roles of IL-5 and eosinophils in the pathophysiology of asthma was also discussed below.

Pathophysiology of Asthma, IL-5, and Eosinophils

Eosinophils are granulocytes that form one to four percent of circulating white cell count. In a normal homeostatic state, almost all eosinophils are found in tissues after they have been released by the CD34+ progenitor cells in the bone marrow [9]. They contain several granules containing major basic protein (MBP), eosinophil-derived neurotoxin (EDN), eosinophil cationic protein (ECP), and eosinophil peroxidase (EPO). When eosinophils are activated, they mediate cellular injury by releasing the contents of these granules. Eosinophils can also release several cytokines, reactive oxygen species, chemokines, and lipid mediators that are pro-inflammatory. During an eosinophilic asthma attack, there is an increased production of eosinophils by the bone marrow. These cells migrate to the airways and once activated, they start secreting the content of their granules [10]. This leads to damage to the smooth muscles of the airways resulting in bronchoconstriction [11]. Eosinophils are also involved in the remodeling of airways. In vivo studies have shown that they can influence the activity of fibroblasts [12]. Bousquet et al. even reported that the levels of eosinophils in blood and bronchoalveolar lavage are associated with the severity of asthma [10]. Levels of eosinophils usually improve after an asthma attack and several studies have proved that the long-term use of glucocorticoids can help lower the level of eosinophils [13].

Interleukin-5 plays an important role in the formation of eosinophils in the bone marrow, as well as in their maturation, migration, and activation. IL-5 also inhibits apoptosis, thus promoting the survival of eosinophils [9,14]. Some studies have suggested that Eotaxin, a chemokine, has a dose-dependent capacity to stimulate eosinophil production by progenitor cells in the bone marrow and the role of eotaxin was independent of IL-5 [15]. However, Foster et al. and Tanaka et al. have also proved that the inhibition of IL-5 or using animals that are deficient in IL-5 receptors can lead to a lower level of eosinophils and less severe remodeling in animals with asthma [16-17]. In humans, the inhalation of IL-5 led to airway hyperreactivity and eosinophilia in asthmatics [18].

Mepolizumab

Mepolizumab is a humanized monoclonal antibody that works against interleukin-5. It is used for the treatment of severe eosinophilic asthma and has been approved for clinical use since 2015. In a study conducted by Bel et al., treatment with mepolizumab lowered the glucocorticoid dose required for severe eosinophilic asthma and the reduction was 2.39 times higher with mepolizumab than with the placebo [19]. In another study by Nair et al., 750 mg mepolizumab each month for five months lowered the prednisone dose by a mean of 83.8±33.4% of their maximum possible dose, as compared with 47.7±40.5% in the placebo group [20]. The drug can decrease the yearly exacerbation rate of asthma (47% and 53% lower with 250 mg intravenous and 250 mg subcutaneous mepolizumab respectively) [21-22] and improve the forced expiratory volume in one second (FEV1) (mean increase of 100 mL with 75mg intravenous mepolizumab and 98 mL with 100 mg subcutaneous mepolizumab) [20,23-24] and peak expiratory flow (PEF) [25]. With the proper inhibition of IL-5, this drug can cause a decrease in the sputum and blood eosinophil count [20,24-30], which can be dose-dependent [31]. Mepolizumab has shown its role in improving the quality of life of asthmatics with an improved Asthma Control Questionnaire (ACQ) and St. George's Respiratory Questionnaire (SGRQ) score [23]. While some studies have shown that the route of administration had no effect on the exposure-response relationship [31], Ortega et al. found that intravenous mepolizumab lowered the rate of exacerbation by 47% while it was 53% lower via the subcutaneous route when compared with the placebo [23]. Mepolizumab also reduced airway eosinophils and decreased the expression of lumican, tenascin, and procollagen III in the bronchial mucosal reticular basement membrane. Tissue remodeling was slowed with an inhibition of eosinophilic expression of transforming growth factor beta one (TGF-β1) [28].

There are multiple adverse effects associated with mepolizumab use but with a low incidence. The most common ones are asthma exacerbation, nasopharyngitis, headache, diarrhea, constipation, eczema, bronchitis, oropharyngeal pain, malaise, migraine, upper respiratory tract infections (URTI), and injection site reactions [19,23-25,27,31-32].

Reslizumab

Reslizumab is a humanized anti-IL-5 monoclonal antibody. The drug binds to circulating IL-5 with high affinity, thus preventing it from binding to IL-5 receptors [33]. Recent clinical trials have shown that a three mg/kg dose of reslizumab given intravenously has a good long-term safety and efficacy, with patients suffering from moderate-to-severe eosinophilic asthma [34]. The pulmonary functions improved in the study (0.090L at week 16 in the reslizumab-naive group, p<0.0001) and the patients had proper asthma control over the course of the study. Another study by Bjermer et al. demonstrated an improvement in mean FEV1 (115 mL with a dose of 0.3mg/kg and 160 mL with three mg/kg), forced vital capacity (FVC) (130mL with three mg/kg), forced expiratory flow at 25%-75% of the pulmonary volume (FEF 25%-75%) (233 mL with three mg/kg) as well as ACQ and Asthma Quality of Life Questionnaire (AQLQ) scores. The patients also had better asthma Symptom Utility Index scores [35]. These findings were supported by other clinical trials, such as the study by Castro et al., which reported that their patients showed mean changes in the ACQ score of -0.7 in the reslizumab group as compared to -0.3 in the placebo group. Their mean changes in FEV1 were also 0.18 and -0.08 L, respectively. Median reductions from the baseline in sputum eosinophils were 95.4% and 38.7%, respectively, in their study [36]. Another study by the same group showed a reduction in the rate of asthma exacerbations with reslizumab therapy over 52 weeks [37]. In a study by Corren et al., a better improvement in FEV1, ACQ (seven), rescue short-acting beta agonists (SABA) use, and FVC were seen when Reslizumab was used in patients with a baseline eosinophil count of ≥400 cells/μL. Patients with a lower eosinophil count showed non-significant changes with the drug in that study [38].

There was no mortality reported linked directly to the use of the drug. The most common adverse effects were asthma, upper respiratory tract infection, sinusitis, bronchitis, and nasopharyngitis.

Benralizumab

Benralizumab is an afucosylated, humanized, monoclonal antibody against the IL-5 receptor alpha that is found on the surface of basophils and eosinophils [39-40]. The drug can help lower the annual exacerbation rates [41-47] and improve the total asthma symptom score [41,46]. However, in smaller doses of two mg, in one study, it decreased the exacerbation by 33% [45], while in another study, it did not cause any significant change [43]. Studies have also shown an improvement in FEV1 when given at 30 mg for 56 weeks [41], 52 weeks [45], 48 weeks [46], and 12 weeks [48] while Nair et al. failed to find any improvement with the same dose over 28 weeks [47]. The drug also led to lower mucosal, sputum, and blood eosinophil counts [44,49-50]. Nair et al. showed that the use of 30 mg of benralizumab over 28 weeks resulted in a significant reduction in oral glucocorticoid doses as well as an improvement of symptoms and exacerbations. Pham et al. noted that with an IL-5 receptor alpha inhibitor like benralizumab, serum IL-5, eotaxin/ CCL11, and eotaxin-2/CCL24 levels were higher compared to placebo [50].

The main side effects associated with benralizumab included nasopharyngitis, injection site reaction, upper respiratory tract infection, and worsening asthma. A small portion of patients in a study conducted by Nowak et al. also experienced pyrexia, tachycardia, and anxiety.

## Conclusions

Over the last two decades, the pharmacological world has taken great initiatives to find new and safer drugs with good efficacy. Mepolizumab was the first monoclonal antibody against IL-5 approved for use in 2015. Many clinical trials have been conducted for new drugs, and reslizumab also gained approval by the Food and Drug Administration (FDA) in 2016 and benralizumab in 2017. This review found that, at present, with the reported clinical trials, there are no major adverse effects that led to any mortality directly caused by these three monoclonal antibodies. However, these drugs have been clinically used for a short period of time and longer follow-ups should be performed to monitor for any more serious adverse effects. At present, prudence should be maintained when using these therapies and any major adverse effects should be reported.
